# Predicting Motor Imagery Performance From Resting-State EEG Using Dynamic Causal Modeling

**DOI:** 10.3389/fnhum.2020.00321

**Published:** 2020-08-06

**Authors:** Minji Lee, Jae-Geun Yoon, Seong-Whan Lee

**Affiliations:** ^1^Department of Brain and Cognitive Engineering, Korea University, Seoul, South Korea; ^2^Department of Artificial Intelligence, Korea University, Seoul, South Korea

**Keywords:** motor imagery, brain-computer interface, dynamic causal modeling, effective connectivity, electroencephalography

## Abstract

Motor imagery-based brain–computer interfaces (MI-BCIs) send commands to a computer using the brain activity registered when a subject imagines—but does not perform—a given movement. However, inconsistent MI-BCI performance occurs in variations of brain signals across subjects and experiments; this is considered to be a significant problem in practical BCI. Moreover, some subjects exhibit a phenomenon referred to as “BCI-inefficiency,” in which they are unable to generate brain signals for BCI control. These subjects have significant difficulties in using BCI. The primary goal of this study is to identify the connections of the resting-state network that affect MI performance and predict MI performance using these connections. We used a public database of MI, which includes the results of psychological questionnaires and pre-experimental resting-state taken over two sessions on different days. A dynamic causal model was used to calculate the coupling strengths between brain regions with directionality. Specifically, we investigated the motor network in resting-state, including the dorsolateral prefrontal cortex, which performs motor planning. As a result, we observed a significant difference in the connectivity strength from the supplementary motor area to the right dorsolateral prefrontal cortex between the low- and high-MI performance groups. This coupling, measured in the resting-state, is significantly stronger in the high-MI performance group than the low-MI performance group. The connection strength is positively correlated with MI-BCI performance (Session 1: *r* = 0.54; Session 2: *r* = 0.42). We also predicted MI performance using linear regression based on this connection (*r-squared* = 0.31). The proposed predictors, based on dynamic causal modeling, can develop new strategies for improving BCI performance. These findings can further our understanding of BCI-inefficiency and help BCI users to lower costs and save time.

## Introduction

Motor imagery-based brain–computer interface (MI-BCI) systems allow users to control computer applications by imagining a movement, without physically performing the muscle activity (Wolpaw et al., 2002). For example, robot arms (Edelman et al., 2019), wheelchairs (Kim et al., 2016), and exoskeletons (Jeong et al., 2020) can be controlled by the user’s brain activity. Thus, these systems have the potential for application in medical fields related to disabled people and motor function rehabilitation. Many researchers have recently sought to expand its application to able-bodied people (Van Gerven et al., 2009; Lee et al., 2016). Generally, MI-BCIs use electroencephalography (EEG) to measure the voluntary modulation of brain rhythms. One of the most representative features is event-related desynchronization/synchronization (ERD/ERS), which reflects a decrease or an increase of oscillatory activity pertaining to events, respectively (Neuper et al., 2006). These changes in brain signals are used as the fundamental characteristics of MI, which measure the power decrease or increase at specific frequencies and in certain brain regions. Many methods have been proposed to improve the performance of MI-BCIs; however, considerable issues must be addressed before MI-BCIs can be practically implemented in real scenarios. The most prominent issue is the inconsistent MI performance that results from the variations in brain signals between different subjects and experiments (Lotte et al., 2007; Lee et al., 2019a). Previous studies have reported that subject performances fluctuate and 15–30% of subjects cannot generate voluntary brain rhythms (Guger et al., 2003; Ahn and Jun, 2015; Sannelli et al., 2019)—a phenomenon known as “BCI-illiteracy” or “BCI-inefficiency” (Sannelli et al., 2019). Therefore, understanding this phenomenon and performance variations is considered an important issue in MI-BCI (Lotte and Jeunet, 2018). In addition, BCI-illiteracy is a methodologically improper concept because it depends on faulty assumptions that BCI users have functional or physiological characteristics that interfere with their skilled BCI performance. Consequently, this term is an inappropriate concept to describe the difficulties that users face when operating a BCI system (Thompson, 2019). In this sense, we use an alternative term, BCI-inefficiency.

Many studies have been performed to find pre-experimental predictors of MI-BCI performance, to save resources and time (Blankertz et al., 2009; Sannelli et al., 2019). Most of these studies can be categorized into either (i) assessing a subject’s condition through psychological questionnaires or (ii) assessing their brain activity by taking EEG measurements directly before the MI experiment. Among the psychological predictors, fatigue is directly related to BCI performance. The feature was extracted using dimension reduction and linear discriminant analysis (LDA) classifier was trained. This BCI performance was compared by quantifying into two groups according to the self-reported rating about fatigue. As a result, BCI performance was significantly high when self-reported fatigue was low during BCI game. Because low fatigue showed effortless control of BCI (Myrden and Chau, 2015). In addition, physical fatigue recorded by physiological changes affected self-reported MI ability. In specific, MI ability was significantly decreased after intermittent exercise (Ferreira et al., 2020). One study surveyed the questionnaire associated with kinesthetic imagery before the MI experiment. Common spatial pattern (CSP) and Fisher LDA were used in a conventional way. Consequently, users’ self-prediction responses to a questionnaire have been reported to correlate with their MI-BCI performance (*r* = 0.64) (Ahn et al., 2018). However, some psychological factors such as fatigue are subjective and therefore are not suitable for describing BCI-inefficiency. In addition, given the length of each training session, limitations still exist in that mental state is unlikely to be consistent overall.

Objective psychological factors such as spatial and visuo-motor coordination abilities also were related to BCI performance. The BCI performance measured from CSP and shrinkage LDA and personality and cognitive profile using psychometric questionnaires were compared. The mental rotation test, which measures spatial ability, showed significantly correlated with BCI performance (*r* = 0.69). However, neurophysiological patterns such as alpha and beta power did not relate to BCI performance (Jeunet et al., 2015, 2016). Similarly, MI performance was calculated using CSP and LDA, and motor skills (*r* = 0.42) and concentration level (*r* = 0.50) were explored as sensorimotor rhythm (SMR) predictors (Hammer et al., 2012). This study focused on pattern recognition rather than human learning for BCI control. So, the next study explored these two psychological factors in the neurofeedback training session. As a result, SMR could only be modulated well by visuo-motor coordination ability, which represents motor skills (*r-squared* = 0.082) in the neurofeedback training session (Hammer et al., 2014). Another study had reported the relationship with age and the average amount of upper limb movement for modulating alpha power associated with BCI. These two factors were positively correlated with the strength of alpha power with 94% confidence using the multiple linear regression (Randolph et al., 2010). The reliable and reproducible predictors of BCI performance contribute to a better understanding of the BCI control. However, these predictors may be less practical because they cannot evaluate and train potential BCI users in a locked-in state, in whose muscular movement is impossible and BCI control is really necessary.

The SMR has been proposed as a neurophysiological indicator (Blankertz et al., 2010), and it is calculated from the mu rhythms (about 9–14 Hz) measured over sensorimotor areas in the C3 and C4 channels in resting-state EEG. These rhythms have shown a significant correlation with MI performance trained using CSP and LDA (*r* = 0.53). Furthermore, higher theta and lower alpha powers were observed in the BCI-inefficiency compared with the BCI-efficient subjects. As a result, this study demonstrated a positive correlation between MI performance using CSP and Fisher LDA and the alpha-theta ratio predictor (*r* = 0.59) (Ahn et al., 2013). Some studies have indicated a relationship between BCI-inefficiency and power spectral density at different frequencies. In particular, gamma oscillations used to infer a subject’s intention have a causal influence on a subject’s BCI capacities. Consequently, BCI performance using spectral power and support vector machine was significantly correlated with predicted BCI accuracy using gamma power (*r* = 0.10) (Grosse-Wentrup et al., 2011). However, these studies predicted MI performance for a single session only. Given the variability of brain signals across different conditions within the same subject, it is necessary to investigate the effects of applying these predictors across various sessions. In addition to the SMR, other EEG features have been proposed to predict MI-BCI performance. Spectral entropy in the C3 channel of eye closed resting-state EEG has been found to correlate with SMR-BCI performance using CSP and LDA in both sessions (Session 1: *r* = 0.61; Session 2: *r* = 0.70) (Zhang et al., 2015a). This predictor can apply for both intra- and inter-session conditions. However, it has not been proven to be applicable to patients such as stroke. In another approach, inter-region connectivity was investigated, not simply the brain activity in a particular region. One study used coherence and phase lag index as the functional connectivity measure. Based on these two measures, network properties were calculated. In the eye closed resting-state, many network properties were directly related to BCI performance. Specifically, mean functional connectivity, node degrees, edge strengths, clustering coefficient, local efficiency, and global efficiency were positively correlated with BCI classification accuracy, whereas the characteristic path length was negatively correlated with BCI classification accuracy. As a primary result, a positive correlation with MI performance using CSP and LDA was observed using a coherence-based clustering coefficient across two sessions (Session 1: *r* = 0.29; Session 2: *r* = 0.42). MI performance was predicted using coherence (except outliers) in Session 2 [root mean square error (RMSE) = 12.2%] (Zhang et al., 2015b). These studies applied the predictor to two sessions and demonstrated that it had a significant correlation with MI performance. However, the relationship was not sufficiently close for the predictor to be employed as an MI-BCI performance indicator in real life applications. Furthermore, these studies have used only one classifier when calculating MI-BCI performance, even though the performance variation depends on both classifier and session. Therefore, a large public database should be used to find possible predictors across a variety of classifiers and sessions, to verify the utility of this MI predictor.

Brain connectivity describes the exchange of information between brain regions (Zhang et al., 2017). The functional connectivity is observable evidence that can be determined as a measure of statistical dependencies. This measure of functional connectivity between the two regions is the same, and it does not indicate directionality (Friston, 2011). However, effective connectivity explains how one region of the brain affects other regions (Lee et al., 2019b). Therefore, it is useful to observe interregional changes in brain networks when investigating certain phenomena. Effective connectivity can be described using a set of common measures that plot directionality between brain regions. For example, there are the following measures: Granger causality, partial directed coherence, and the direct transfer function (Sakkalis, 2011). Above all, dynamic causal modeling (DCM) reflects inferences about the couplings between brain regions/sources and is based on a Bayesian approach (Kiebel et al., 2008). As a consequence, in contrast to functional connectivity and some causal model, DCM needs a defined *a priori* knowledge and hypothesis-driven models (Kasess et al., 2010). This Bayesian approach directly assesses the posterior probability distribution of the estimated model parameters, given measured EEG data and a specific priori model at the single-subject level. For group-level analysis of model parameters, this approach based on fixed-effects analysis as the inference method has the advantage that the precisions of the subject-specific multivariate parameter estimates are considered (Chen et al., 2008; Kasess et al., 2010; Bönstrup et al., 2016). This approach compares various hypothesis-based models and helps to select an optimal specific model. Furthermore, volume conduction—a problem for the conventional measurement methods—can be avoided by including the source reconstruction to assess directionality between brain regions (Lee et al., 2019b).

Many studies have used DCM to investigate the connections between brain regions during MI. In a DCM study using functional magnetic resonance imaging (fMRI), a forward connection was found between the supplementary motor area (SMA) and the primary motor cortex (M1). In particular, the SMA exhibited a strong suppressive influence on M1 during MI (Kasess et al., 2008). In addition, by using a combined fMRI and EEG approach, the coupling between SMA and M1 was shown to contain significant information for MI (Bönstrup et al., 2016). The SMA is considered to be the main active region in MI generation and is involved in the preparation of movements (Kuhtz-Buschbeck et al., 2003). Recent studies have shown that effective connectivity is similar under motor execution (ME) and MI tasks through DCM; furthermore, these networks have been reported to include the dorsolateral prefrontal cortex (DLPFC) and premotor cortex (PMC) in addition to the SMA and M1 (Kim et al., 2018). These brain regions are necessary to generate the rich MI sources used to control BCIs (Hochberg et al., 2006; Aflalo et al., 2015). The PMC exhibits overlapping between active and peripheral regions during ME and MI, and it is employed in language production, movement observation, and action recognition (Lotze and Halsband, 2006). The DLPFC is closely connected with the cortical control of movement and may be linked with the SMA (Middleton and Strick, 1994). In this regard, certain brain regions—though not directly related to the motor cortex—can be associated with MI.

In this study, we investigate the correlations between MI-BCI performance and the subject’s resting-state network before the BCI experiment takes place. DCM was used to explore the effective connectivity between two regions with directionality. In particular, we considered the DLPFC in addition to the conventional sensorimotor areas as the DCM region of interest (ROI). We assessed the subjects’ psychological questionnaires and band powers (from their resting-state EEG) before the MI experiment, for comparison with previous studies. We hypothesized that the coupling strength in the motor network constructed using DCM would be correlated with the MI-BCI performance. Finally, using linear regression, we predicted the MI-BCI performance with the proposed coupling strength. These findings could help build an understanding of the MI mechanism and improve overall MI-BCI performances by investigating the characteristics of poorly performing subjects.

## Materials and Methods

### EEG Dataset

We used a public EEG dataset from GigaDB (Lee et al., 2019a). These data contain EEG signals measured during MI experiments focusing on left and right hand grasping motions. The subjects’ psychological and physical conditions were surveyed using questionnaires and 1 min eye-open resting-state EEG data were recorded before the MI experiments. The experiments were conducted over two sessions, which took place on different days. The data comprised 54 healthy subjects (24.8 ± 3.8 years; 25 females). Among the subjects, 38 were naive BCI users and the remainder had previous experience. EEG signals were recorded using 62 Ag/AgCl electrodes.

### MI-BCI Performance and Group Categorization

The EEG signals were processed using the OpenBMI toolbox (Lee et al., 2019a); the data were band-pass filtered between 8 and 30 Hz—the frequency band relevant to motor movements. A 5th order Butterworth filter was used for all band-pass filter analyses; next, the continuous EEG signals were segmented from 1 to 3.5 sec (measured from stimulus onset) (Pfurtscheller and Neuper, 2001). Moreover, 20 channels were selected in the motor cortex region (FC1, FC2, FC3, FC4, FC5, FC6, Cz, C1, C2, C3, C4, C5, C6, CPz, CP1, CP2, CP3, CP4, CP5, and CP6).

We used several popular methods to calculate MI performance (Lee et al., 2019a). We extracted four features, as follows: (i) CSP (Ramoser et al., 2000)—a spatial pattern that maximizes the discrimination of the binary classes; (ii) common spatio-spectral pattern (CSSP) (Lemm et al., 2005)—a pattern using spectral information based on CSP; (iii) filter bank common spatial pattern (FBCSP) (Ang et al., 2012)—a pattern using optimal spatio-spectral filters based on a filter bank composed of several frequency bands; and (iv) Bayesian spatio-spectral filter optimization (BSSFO) (Suk and Lee, 2012)—a pattern using subject-dependent frequency bands within the Bayesian framework. For the classifier, LDA was used to decode the left or right hand imagery. Each experimental task comprised a training phase and a testing phase. To validate the MI performance, ten-fold cross-validation was used for all data (training + testing data) (CSP-cv). In summary, we achieved the MI-BCI performance with CSP-cv, CSP, CSSP, FBCSP, and BSSFO.

To compare the MI performance against the resting-state EEG, we divided them into two performance groups: high (good MI performance group) and low (poor MI performance group). The median performance in each five performance according to classifiers was used to separate the subjects into high- or low-MI performance groups (Zhang et al., 2016).

### Relationship With MI-BCI Performance

#### Questionnaire Scores

We took seven response fields from the pre-experimental questionnaire: comfort, motivation, concentration, eye fatigue, drowsiness, physical condition, and mental condition. These items were graded on a Likert scale from 1 to 5. For “comfort,” 1 signified relaxation, and 5 signified anxiety. Under “motivation,” 1 indicated excitement, and 5 indicated boredom. In the “concentration,” “eye fatigue,” “drowsiness,” “physical condition,” and “mental condition,” 1 and 5 indicated very good and very bad or tired in intensity level, respectively.

#### Band Power of Resting-State EEG

We calculated the average power of the EEG signals, to decompose them into functionally distinct frequency bands. We further divided them into five regions: frontal (Fp1-2, AF3-4, AF7-8, AFz, F3-4, F7-8, and Fz), sensorimotor (FC1-6, C1-6, Cz, CP1-6, and CPz), temporal (FT9-10, T7-8, and TP7-10), parietal (P1-4, P7-8, PO3-4, and POz), and occipital (O1-2, Oz, and PO9-10) regions (Supplementary Figure S1). At the sensor level, EEG signals were averaged according to five different cortical regions. The band powers were also measured for the delta (1–4 Hz), theta (4–8 Hz), alpha (8–15 Hz), beta (15–25 Hz), and gamma (25–40 Hz) bands (Ahn et al., 2013).

#### Dynamic Causal Modeling of Resting-State EEG

##### Pre-processing

The continuous EEG signals were pre-processed using the EEGLAB toolbox (Delorme and Makeig, 2004) based on MATLAB. Data from 56 channels across the scalp surface (using the international 10–10 system) were obtained to implement DCM (Lee et al., 2017). The resting-state EEG was band-pass filtered in the 4–45 Hz (Van de Steen et al., 2019). The delta band in the 1–4 Hz range was excluded because, unlike other frequency bands, it can be contaminated relatively easily by artifacts such as eyeball movement and blinking (Ahn et al., 2013). The continuous 1 min EEG data were segmented from 1 sec without overlap (Van de Steen et al., 2019). Then, the eye-blink correction was manually performed using infomax, which is one of the most widely used independent component analysis algorithm to minimize the artifacts. Finally, the epoched data were average-referenced.

##### 3D source reconstruction

We used the statistical parametric mapping (SPM) toolbox in MATLAB (Litvak et al., 2011). In the 3D channel location information, EEG channel locations were transformed to match the template head. This head model was assigned to all subjects using 3D coordinate values. The boundary element method (BEM) was used for building a head model (forward model, mapping source signals to sensor signals). Each source was also modeled by a single equivalent current dipole (ECD) (Kiebel et al., 2006) for reconstructing sources (inverse model, mapping sensor signals to the source signals). To estimate the cortical sources, the inversion index was set to 1 to trace different types of forward models and inverse solutions. Mesh resolution can be maintained at normal (approximately 4,000 vertices per hemisphere).

##### DCM specification

**FIGURE 1:**
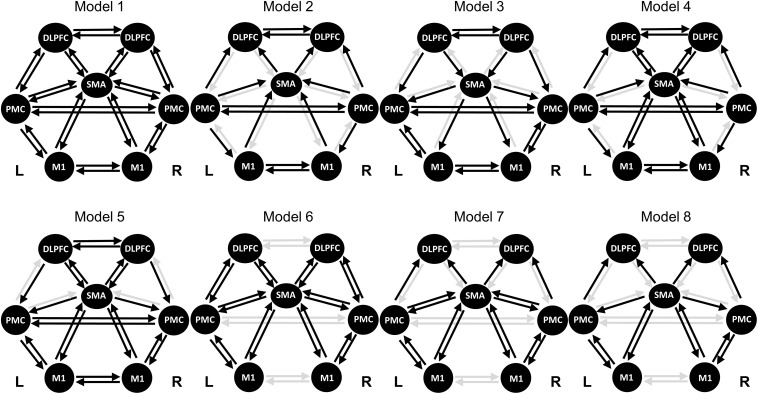
Model specifications of effective connectivity based on the dynamic causal model. The regions of interest (ROIs) consist of the SMA, left/right PMC, left/right M1, and left/right DLPFC. The resting-state has no external stimulus, thus no input is specified. The selected connection for each model is indicated by a black arrow. SMA, supplementary motor area; PMC, premotor cortex; M1, primary motor cortex; DLPFC, dorsolateral prefrontal cortex.

M1, SMA, and PMC are well known to be linked to MI (Kasess et al., 2008; Begliomini et al., 2015; Bönstrup et al., 2016). Recently, the role of DLPFC in MI has been revealed (Kim et al., 2018). Therefore, we selected the seven ROIs: SMA, left/right M1, left/right PMC, and left/right DLPFC. We also employed the Montreal Neurological Institute (MNI) coordinates for both side regions, based on the source locations reported in previous work (Kim et al., 2018). Table 1 lists the MNI coordinates for seven ROIs. The prefrontal-dependent regions were reported to have no physiologically specific interactions with the M1 (Luppino et al., 1993; Rizzolatti and Luppino, 2001). Therefore, we excluded the connection between DLPFC and M1 and finally organized the eight DCM models (Figure 1). In addition, for the resting-state, we did not select an input from the neural model because no external input exists.

**TABLE 1:** Montreal Neurological Institute (MNI) coordinates for location information.

**ROI**	**MNI coordinates**		
	***x***	***y***	***z***
SMA	0	−4	65
Left M1	−38	−26	53
Right M1	38	−26	53
Left PMC	−48	−15	50
Right PMC	48	−15	50
Left DLPFC	−42	40	25
Right DLPFC	42	40	25

*ROI, region of interest; SMA, supplementary motor area; M1, primary motor cortex; PMC, premotor cortex; DLPFC, dorsolateral prefrontal cortex.*

Dynamic causal modeling uses a neural mass model to explain the source activity of EEG signals (David and Friston, 2003). The model imitates the source activity by using three neural sublayers assigned to the three cortical layers, namely the granular sublayer, the supra-granular sublayer, and the infra-granular sublayer. This model has hierarchical features; forward connections start in the infra-granular layer and end in the granular layer and backward connections link agranular layers (Garrido et al., 2007). All cortico-cortical connections are excitatory, so the DCM can be identified in neuronal state equation by average synaptic dynamics in each sublayer.

(1) $$\dot{x}=f\,\left(x,u,\theta \right)$$

where $\dot{x}$ indicates the evolution of neuronal state *x* parameterized by θ of the state and input *u*.

(2) $$y=L\,\left(\theta \right)\,x_{0}+\varepsilon$$

where *x*_*0*_ is output of specific states and *L*(*θ*) indicates the local field indicating the location and orientation of sources (i.e., spatial forward model). In specific, θ includes the parameters for forward and backward connections (coupling strength). The ε refers to observation error. Finally, EEG signals *y* connects the neuronal states to observed EEG channel data (Kiebel et al., 2006).

##### Bayesian model selection (BMS)

Bayesian model selection (BMS) is an effective method of deciding the most likely set of competitive hypotheses for the models that generated the observed data (Stephan et al., 2009). We applied BMS averaging with fixed-effects analysis to determine the most likely model given the data.

The inversion of a particular DCM, *m*, coincides with an approximation of the posterior probability on the several models.

(3) p⁢(θ|y,m)∝p⁢(y|θ,m)⁢p⁢(θ|m)

This approximation uses the Bayes factor based on Expectation-Maximization algorithm. This aims to minimize the free energy *F* = −ln *p*(*y*|*m*) as the negative marginal log-likelihood. Then, the variational Bayes factor is used as an approximation and the log-evidence is used for model comparison. Finally, the best model is the highest log-evidence ln *p*(*y*|*m*) (Garrido et al., 2007). In our study, eight DCM models were estimated and one was selected using BMS.

### Statistical Analysis

We first performed the one-way analysis of variance (ANOVA) to investigate the differences in MI performance using CSP-cv, CSP, CSSP, FBCSP, and BSSFO. Next, the correlation was used to verify that the MI performance between the two sessions was similar. To investigate the differences in resting-state EEG between the high- and low-MI performance groups, we performed the two-way ANOVA (session × group). In all ANOVA, the two-sample *t*-test was used with Bonferroni correction for multiple comparisons as *post hoc* analysis. Pearson’s correlation was also used to examine the relationship between MI performance and resting-state EEG. Similarly, Bonferroni correction was applied to correlation analysis for multiple comparisons. For the questionnaire and band power, we used only the MI performance measured by CSP-cv for a fair comparison with previous studies (Ahn et al., 2013, 2018; Zhang et al., 2015b). We also predicted the MI performance based on significantly selected coupling strength, by applying linear regression to the MI-BCI performance in the resting-state. The 10-fold cross-validation was used to prevent overfitting (Lever et al., 2016). Then, we evaluated the predicted MI-BCI performance compared with the actual MI-BCI performance based on CSP-cv, CSP, CSSP, FBCSP, and BSSFO, using the *r*-squared and RMSE, where *r*-squared is a statistical value of how close the data are to the fitted regression line, and RMSE is a measure of the difference between the actual and the predicted MI-BCI performance (Varatharajan et al., 2018).

## Results

### Differences in MI Performance

Using CSP-cv, CSP, CSSP, FBCSP, and BSSFO, we observed a significantly positive correlation of the two-class MI performances between two sessions on different days (CSP-cv: *r* = 0.986, *p* < 0.001; CSP: *r* = 0.988, *p* < 0.001; CSSP: *r* = 0.993, *p* < 0.001; FBCSP: *r* = 0.993, *p* < 0.001; BSSFO: *r* = 0.993, *p* < 0.001). We also investigated the differences in MI-BCI performances using five methods within each session. No significant differences in MI performances using five methods with Bonferroni correction were observed in both sessions [Session 1: *F*_(4,265)_ = 0.22, *p* = 0.929; Session 2: *F*_(4,265)_ = 0.33, *p* = 0.859].

**FIGURE 2:**
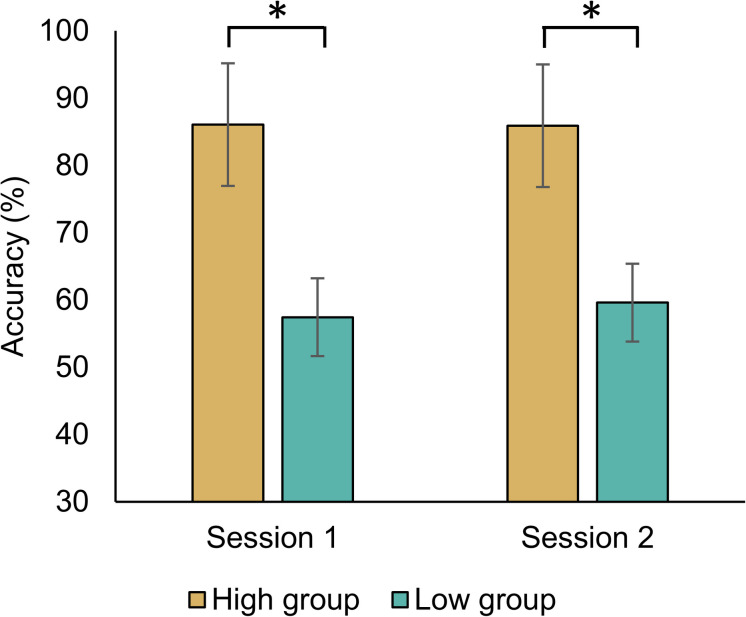
Averaged MI classification performance based on CSP-cv for both sessions. The group was divided based on the median of MI classification accuracy across all subjects. The *p*-values below 0.05 are highlighted by an asterisk.

We divided the high- and low-MI groups in each classifier. There was no significant difference in MI performance with Bonferroni correction according to session and group using five methods (Table 2). Nevertheless, we compared the MI performance of each group because the classification accuracy in high-MI group was all higher than that in the low-MI group by dividing each group based on the median of the performance of all subjects. For MI-BCI performances based on the CSP-cv, five subjects were displaced from a low-MI group to a high-MI group; moreover, two subjects were displaced from a high-MI group to a low-MI group between Sessions 1 and 2. The significant differences in MI performance observed between the high- and low-MI groups were explored using CSP-cv [Session 1: *t*_(52)_ = 14.125, *p* < 0.001; Session 2: *t*_(52)_ = 12.115, *p* < 0.001] (Figure 2). As expected, the MI classification accuracy for the higher group was greater than that for the lower group during both sessions. Supplementary Figure S2 shows the MI performances in high- and low-MI groups for CSP, CSSP, FBCSP, and BSSFO. Similar to CSP-cv, there were significant differences observed in MI performance across the other four classifiers [CSP – Session 1: *t*_(52)_ = 16.323, *p* < 0.001, Session 2: *t*_(52)_ = 13.094, *p* < 0.001; CSSP – Session 1: *t*_(52)_ = 17.833, *p* < 0.001, Session 2: *t*_(52)_ = 15.341, *p* < 0.001; FBCSP – Session 1: *t*_(52)_ = 19.320, *p* < 0.001, Session 2: *t*_(52)_ = 15.509, *p* < 0.001; BSSFO – Session 1: *t*_(52)_ = 19.777, *p* < 0.001, Session 2: *t*_(52)_ = 18.961, *p* < 0.001]. In addition, 7, 5, 6, and 7 subjects changed from the low- to high-MI group between Sessions 1 and 2 under CSP, CSSP, FBCSP, and BSSFO, respectively; conversely, 3, 4, 6, and 5 subjects changed from the high- to low-MI group on different days under CSP, CSSP, FBCSP, and BSSFO, respectively. To summarize, the MI classification accuracy for the low-MI group tended to not exceed 60%, whereas the high-MI group showed an average classification accuracy greater than 80%.

**TABLE 2:** Statistical results for the differences in MI performance.

**Classifier**	**Session**			**Group**			**Session × Group**		
	**dof**	***F***	***p-*value**	**dof**	***F***	***p-*value**	**dof**	***F***	***p-*value**
CSP_cv	1	0.40	0.527	1	2.28	0.133	1	0.09	0.768
CSP	1	0.16	0.690	1	1.87	0.174	1	0.71	0.401
CSSP	1	0.16	0.692	1	0.81	0.369	1	0.04	0.835
FBCSP	1	0.16	0.694	1	1.51	0.221	1	0.02	0.899
BSSFO	1	0.20	0.658	1	0.29	0.592	1	0.24	0.626

*The session factor indicates Session 1 and Session 2, whereas the group factor indicates high-MI performance group and low-MI performance group. The session × group represents the interaction between session and group factors. dof, degree of freedom; CSP_cv, common spatial pattern with cross-validation; CSP, common spatial pattern; CSSP, common spatio-spectral pattern; FBCSP, filter bank common spatial pattern; BSSFO, Bayesian spatio-spectral filter optimization.*

### Relationship With Questionnaire Score

We investigated differences in questionnaire score between the high- and low-MI groups based on CSP-cv (Table 3). There were no significant differences observed in any score according to session and group with Bonferroni correction. In addition, we calculated the correlation with MI-BCI performance (Table 4). Similarly, no significant correlation with MI-BCI performance with Bonferroni correction was found in either session.

**TABLE 3:** Statistical results for the differences in questionnaire scores according to MI performance using CSP-cv.

**Questionnaire**	**Session**			**Group**			**Session × Group**		
	**dof**	***F***	***p*-value**	**dof**	***F***	***p*-value**	**dof**	***F***	***p*-value**
Comfort	1	0.01	0.963	1	2.28	0.134	1	0.89	0.348
Motivation	1	0.01	0.922	1	0.16	0.692	1	0.01	0.906
Concentration	1	0.08	0.782	1	1.03	0.312	1	0.62	0.434
Eye fatigue	1	0.36	0.552	1	0.08	0.784	1	0.27	0.607
Drowsiness	1	0.04	0.838	1	0.50	0.481	1	0.20	0.655
Physical condition	1	0.28	0.597	1	2.80	0.097	1	2.40	0.124
Mental condition	1	0.64	0.423	1	0.49	0.486	1	0.26	0.608

*The questionnaires consisted of seven questionnaires and were taken for each session before the MI experiment took place. The session factor indicates Session 1 and Session 2, whereas the group factor indicates high-MI performance group and low-MI performance group. The session × group represents the interaction between session and group factors. dof, degree of freedom.*

**TABLE 4:** Statistical results for correlation in questionnaire scores according to MI performance using CSP-cv.

**Questionnaire**	**Session 1**		**Session 2**	
	***r*-value**	***p*-value**	***r*-value**	***p-*value**
Comfort	0.043	0.758	–0.147	0.295
Motivation	0.042	0.764	–0.002	0.990
Concentration	–0.020	0.888	–0.154	0.271
Eye fatigue	0.089	0.520	0.020	0.886
Drowsiness	–0.133	0.379	–0.076	0.588
Physical condition	–0.096	0.490	–0.197	0.158
Mental condition	–0.144	0.300	–0.039	0.784

*The questionnaires consisted of seven questions and were taken for each session before the MI experiment took place.*

### Relationship With Band Power

Table 5 summarizes the statistical differences in band power between the high- and low-MI groups according to session and group. As a result, the beta power in the sensorimotor region showed a significant difference between the high- and low-MI groups in Session 1 with Bonferroni correction [*t*_(52)_ = 2.67, *p* = 0.009]. However, no significant power differences in other frequency bands were found between the two groups in either session. In addition, an only positive correlation was observed between theta power in the parietal region and MI-BCI performance based on CSP-cv (Table 6).

**TABLE 5:** Statistical results for the differences in band power according to MI performance using CSP-cv.

**Region**	**Frequency**	**Session**			**Group**			**Session × Group**		
		**dof**	***F***	***p*-value**	***dof***	***F***	***p*-value**	***dof***	***F***	***p*-value**
Frontal	Delta	1	3.24	0.074	1	4.27	**0.041**	1	0.85	0.357
	Theta	1	0.20	0.655	1	0.96	0.338	1	0.24	0.624
	Alpha	1	0.45	0.505	1	0.01	0.942	1	0.77	0.383
	Beta	1	0.06	0.814	1	0.75	0.389	1	0.46	0.497
	Gamma	1	0.69	0.406	1	0.05	0.825	1	0.66	0.418
Sensorimotor	Delta	1	1.08	0.301	1	2.20	0.141	1	0.01	0.950
	Theta	1	0.36	0.552	1	2.12	0.148	1	0.09	0.762
	Alpha	1	0.01	0.919	1	2.64	0.107	1	0.01	0.965
	Beta	1	0.30	0.583	1	5.58	**0.020**	1	0.39	0.533
	Gamma	1	1.09	0.299	1	1.39	0.241	1	0.01	0.996
Temporal	Delta	1	0.45	0.504	1	1.15	0.286	1	0.13	0.715
	Theta	1	0.19	0.661	1	1.74	0.189	1	0.13	0.719
	Alpha	1	0.01	0.963	1	0.52	0.472	1	0.11	0.745
	Beta	1	0.05	0.822	1	0.88	0.349	1	1.80	0.182
	Gamma	1	0.32	0.573	1	0.44	0.506	1	1.38	0.243
Parietal	Delta	1	0.95	0.330	1	0.77	0.381	1	0.06	0.813
	Theta	1	0.64	0.426	1	0.36	0.551	1	0.07	0.790
	Alpha	1	0.01	0.912	1	0.03	0.856	1	0.04	0.835
	Beta	1	0.03	0.863	1	2.72	0.102	1	0.34	0.563
	Gamma	1	0.13	0.717	1	0.72	0.399	1	0.53	0.467
Occipital	Delta	1	0.76	0.386	1	0.28	0.596	1	0.14	0.705
	Theta	1	0.26	0.608	1	0.04	0.837	1	0.06	0.805
	Alpha	1	0.04	0.847	1	0.77	0.383	1	0.02	0.877
	Beta	1	0.08	0.777	1	0.01	0.969	1	0.80	0.373
	Gamma	1	0.01	0.996	1	0.01	0.911	1	0.51	0.476

*The brain region was divided into five sub-regions, and the frequency was also divided into five bands as follows: delta (1–4 Hz), theta (4–8 Hz), alpha (8–15 Hz), beta (15–25 Hz), and gamma (25–40 Hz) bands. The session factor indicates Session 1 and Session 2, whereas the group factor indicates high-MI performance group and low-MI performance group. The session × group represents the interaction between session and group factors. The p-values below 0.05 are highlighted in bold. dof, degree of freedom.*

**TABLE 6:** Statistical results for band power correlations according to MI performance using CSP-cv.

**Region**	**Frequency**	**Session 1**		**Session 2**	
		***r*-value**	***p*-value**	***r*-value**	***p-*value**
Frontal	Delta	0.130	0.350	0.219	0.111
	Theta	0.075	0.589	0.225	0.102
	Alpha	0.032	0.817	0.231	0.093
	Beta	0.014	0.920	0.233	0.089
	Gamma	–0.018	0.896	0.221	0.109
Sensorimotor	Delta	–0.022	0.876	0.089	0.523
	Theta	0.067	0.633	0.093	0.503
	Alpha	0.048	0.732	0.077	0.580
	Beta	–0.039	0.781	0.078	0.575
	Gamma	–0.050	0.719	0.033	0.813
Temporal	Delta	–0.108	0.438	0.147	0.289
	Theta	0.224	0.104	0.246	0.073
	Alpha	0.064	0.645	0.136	0.325
	Beta	0.057	0.680	0.068	0.625
	Gamma	–0.004	0.980	–0.022	0.874
Parietal	Delta	0.130	0.349	0.180	0.193
	Theta	0.272	0.047*	0.106	0.446
	Alpha	0.132	0.341	–0.029	0.837
	Beta	0.147	0.289	0.134	0.335
	Gamma	0.070	0.613	–0.038	0.786
Occipital	Delta	0.192	0.165	0.106	0.445
	Theta	0.137	0.323	0.025	0.855
	Alpha	0.097	0.486	–0.046	0.742
	Beta	0.047	0.737	0.057	0.680
	Gamma	0.035	0.800	0.031	0.827

*The brain region was divided into five sub-regions, and the frequency was also divided into five bands as follows: delta (1–4 Hz), theta (4–8 Hz), alpha (8–15 Hz), beta (15–25 Hz), and gamma (25–40 Hz) bands. The p-values below 0.05 are highlighted in bold. ^∗^ with no correction.*

### Relationship With Coupling Strength Based on DCM

For Session 1, Model 4 was chosen through BMS and the connectivity strengths of 20 connections were calculated. For Session 2, Model 2 was determined as a suitable model; this model included 16 connections.

#### Difference Between High- and Low-MI Performance Groups

**FIGURE 3:**
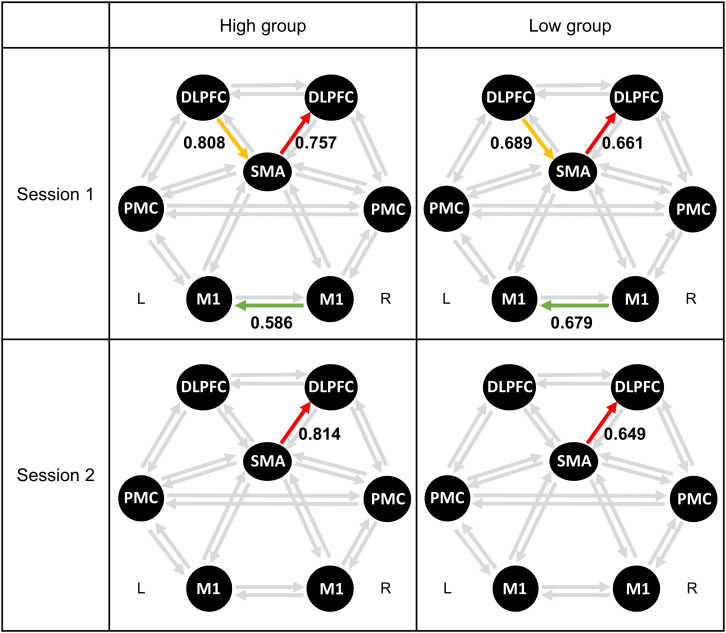
Averaged connectivity strength in resting-state EEG between high- and low-MI performance groups based on CSP-cv. Each colored arrow represents a connection with a significant difference between the high- and low-MI performance groups. SMA, supplementary motor area; PMC, premotor cortex; M1, primary motor cortex; DLPFC, dorsolateral prefrontal cortex.

Table 7 lists the differences across 20 connections between high- and low-MI performance groups for MI-BCI performance, based on CSP-cv. Figure 3 shows the significant connectivity strength between the two groups in each session, based on CSP-cv. In particular, the coupling strength from the SMA to the right DLPFC in the high-MI group was significantly higher than in the low-MI group in both sessions [Session 1: *t*_(52)_ = 2.71, *p* = 0.008 with Bonferroni correction; Session 2: *t*_(52)_ = 4.31, *p* < 0.001 with Bonferroni correction]. Additionally, in Session 1, a higher coupling strength from left DLPFC to SMA was observed in the high-MI group [*t*_(52)_ = 2.76, *p* = 0.008 with Bonferroni correction], whereas a lower coupling strength from right M1 to left M1 was observed in the high-MI group compared with the low-MI group [*t*_(52)_ = −2.78, *p* = 0.009 with Bonferroni correction]. In addition, the differences in coupling strength between high- and low-MI groups based on CSP (Supplementary Table S1), CSSP (Supplementary Table S2), FBCSP (Supplementary Table S3), and BSSFO (Supplementary Table S4) are listed. As with CSP-cv, the coupling strength from the SMA to the right DLPFC in the high-MI group was higher than in the low-MI group in two sessions based on four classifiers in both Session 1 [CSP: *t*_(52)_ = 3.26, *p* = 0.001 with Bonferroni correction; CSSP: *t*_(52)_ = 3.96, *p* < 0.001 with Bonferroni correction; FBCSP: *t*_(52)_ = 2.93, *p* = 0.005 with Bonferroni correction; BSSFO: *t*_(52)_ = 2.76, *p* = 0.008 with Bonferroni correction] and Session 2 [CSP: *t*_(52)_ = 2.90, *p* = 0.005 with Bonferroni correction; CSSP: *t*_(52)_ = 2.91, *p* = 0.005; FBCSP: *t*_(52)_ = 2.76, *p* = 0.008 with Bonferroni correction; BSSFO: *t*_(52)_ = 2.46, *p* = 0.017 with Bonferroni correction]. Similarly, differences in coupling strength from left DLPFC to SMA between two MI groups were observed in Session 1 based on four methods [CSP: *t*_(52)_ = 3.40, *p* = 0.001 with Bonferroni correction; CSSP: *t*_(52)_ = 2.64, *p* = 0.010 with Bonferroni correction; FBCSP: *t*_(52)_ = 3.12, *p* = 0.002 with Bonferroni correction; BSSFO: *t*_(52)_ = 3.07, *p* = 0.003 with Bonferroni correction]. However, there was significant difference in coupling strength from right M1 to left M1 in Session 1 using four classifiers, and in Session 2, strength from left PMC and left DLPFC showed the significant differences between two groups only in CSP [*t*_(52)_ = −2.55, *p* = 0.013 with Bonferroni correction].

**TABLE 7:** The statistical differences in effective connectivity between high- and low-MI performance groups based on CSP-cv.

**ROI**		**Session**			**Group**			**Session × Group**		
**From**	**To**	**dof**	***F***	***p*-value**	**dof**	***F***	***p*-value**	**dof**	***F***	***p*-value**
lM1	SMA	1	1.58	0.211	1	0.48	0.489	1	0.52	0.474
rM1	SMA	1	0.78	0.380	1	0.39	0.534	1	0.01	0.942
lPMC	SMA	1	1.72	0.192	1	1.42	0.236	1	0.10	0.747
rPMC	SMA	1	0.98	0.324	1	0.62	0.434	1	0.01	0.991
lDLPFC	SMA	0	0	NaN	1	7.62	**0.008**	0	0	NaN
rDLPFC	SMA	0	0	NaN	1	0.45	0.506	0	0	NaN
SMA	lM1	0	0	NaN	1	0.56	0.455	0	0	NaN
rM1	lM1	1	3.43	0.067	1	4.79	**0.030**	1	1.38	0.242
lPMC	lM1	1	1.34	0.250	1	2.65	0.106	1	0.69	0.409
SMA	rM1	0	0	NaN	1	0.04	0.842	0	0	NaN
lM1	rM1	1	0.88	0.350	1	0.03	0.861	1	0.01	0.973
rPMC	rM1	1	0.99	0.322	1	1.06	0.306	1	0.01	0.941
rPMC	lPMC	1	3.53	0.062	1	0.36	0.547	1	0.14	0.709
lPMC	rPMC	1	0.42	0.517	1	0.23	0.634	1	0.64	0.423
SMA	lDLPFC	1	0.51	0.477	1	0.02	0.898	1	0.37	0.542
lPMC	lDLPFC	1	0.14	0.704	1	2.33	0.130	1	2.47	0.119
rDLPFC	lDLPFC	1	0.35	0.552	1	0.08	0.780	1	0.06	0.813
SMA	rDLPFC	1	0.74	0.391	1	25.01	**<0.001**	1	1.66	0.200
rPMC	rDLPFC	1	0.13	0.714	1	0.29	0.593	1	0.99	0.323
lDLPFC	rDLPFC	1	1.59	0.210	1	0.21	0.648	1	0.01	0.974

*In Session 1, Model 4 is selected and there are 20 connections. In Session 2, Model 2 is selected and there are 16 connections. Therefore, four connections in Session 2 are excluded (‘NaN’). The session factor indicates Session 1 and Session 2, whereas the group factor indicates high-MI performance group and low-MI performance group. The session × group represents the interaction between session and group factors. The p-values below 0.05 are highlighted in bold. ROI, region of interest; dof, degree of freedom; l/rM1, left/right primary motor cortex; l/rPMC, left/right pre-motor cortex; l/rDLPFC, left/right dorsolateral prefrontal cortex; SMA, supplementary motor area.*

#### Correlation With MI Performance

**FIGURE 4:**
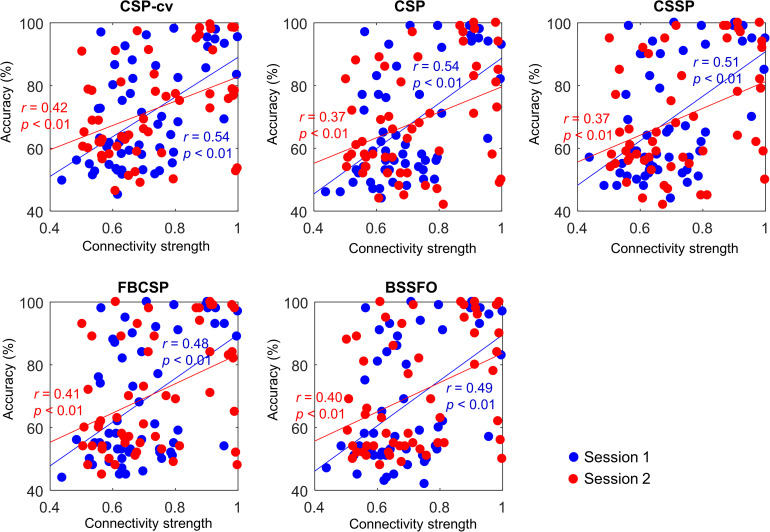
Correlation between connectivity strength from SMA to right DLPFC and MI-BCI performance. Each colored dot represents an individual connectivity strength from SMA to right DLPFC and MI performance. Blue and red indicate Session 1 and Session 2, respectively. SMA, supplementary motor area; DLPFC, dorsolateral prefrontal cortex; CSP-cv, common spatial pattern with cross-validation; CSP, common spatial pattern; CSSP, common spatio-spectral pattern; FBCSP, filter bank common spatial pattern; BSSFO, Bayesian spatio-spectral filter optimization.

To verify the reliability of the proposed predictors, we investigated their correlations with MI-BCI performance. Table 8 lists the correlations between 20 connections in a resting-state EEG and MI-BCI performance, based on CSP-cv. Positive correlation in connectivity strength from the SMA to right DLPFC with Bonferroni correction was observed in both sessions. In Session 1, strength from the left DLPFC to SMA was positively correlated with the MI-BCI performance. Furthermore, the strength from the left PMC to left DLPFC was negatively correlated with MI performance in Session 2. However, there was no correlation with the directionality from right M1 to left M1 that had significant differences between the two MI groups in Session 1. Similar results to those obtained under CSP-cv were obtained when assessing MI performance with CSP, CSSP, FBCSP, and BSSFO (Supplementary Table S5). In particular, the coupling strength from the SMA to right DLPFC was significant in both sessions, for all classifiers. Thus, we depicted the correlation between coupling from SMA to right DLPFC and MI-BCI performance through five methods (Figure 4). In both sessions, this coupling strength was significantly correlated. In Session 1, strength from left DLPFC to SMA was correlated with MI-BCI performance using five methods (Supplementary Figure S3).

**TABLE 8:** Correlations between connectivity strength and MI performance based on CSP-cv.

**ROI**		**Session 1**		**Session 2**	
**From**	**To**	***r*-value**	***p*-value**	***r*-value**	***p*-value**
lM1	SMA	0.054	0.698	0.018	0.898
rM1	SMA	–0.084	0.546	0.034	0.805
lPMC	SMA	–0.193	0.163	–0.177	0.200
rPMC	SMA	–0.076	0.584	–0.069	0.618
lDLPFC	SMA	0.381	0.005*	NaN	NaN
rDLPFC	SMA	–0.037	0.791	NaN	NaN
SMA	lM1	–0.117	0.398	NaN	NaN
rM1	lM1	–0.217	0.115	–0.082	0.554
lPMC	lM1	–0.173	0.212	–0.104	0.453
SMA	rM1	0.010	0.940	NaN	NaN
lM1	rM1	–0.055	0.694	–0.052	0.708
rPMC	rM1	0.091	0.513	0.058	0.675
rPMC	lPMC	0.175	0.206	–0.030	0.831
lPMC	rPMC	0.073	0.599	0.076	0.586
SMA	lDLPFC	0.010	0.943	–0.071	0.610
lPMC	lDLPFC	–0.061	0.659	–0.331	0.014*
rDLPFC	lDLPFC	0.117	0.398	–0.211	0.125
SMA	rDLPFC	0.536	<0.001**	0.419	0.002**
rPMC	rDLPFC	–0.020	0.883	0.154	0.266
lDLPFC	rDLPFC	0.036	0.795	–0.017	0.906

In Session 1, Model 4 is selected and there are 20 connections. In Session 2, Model 2 is selected and there are 16 connections. Therefore, four connections in Session 2 are excluded (‘NaN’). The p-values below 0.05 are highlighted in bold. ROI, region of interest; l/rM1, left/right primary motor cortex; l/rPMC, left/right pre-motor cortex; l/rDLPFC, left/right dorsolateral prefrontal cortex; SMA, supplementary motor area. * with no correction and **<Bonferroni correction.

### Prediction of MI Performance Using Coupling Strength

We predicted MI-BCI performance using the coupling strength from SMA to right DLPFC in resting-state EEG. Table 9 shows the *r*-squared and RMSE values between the predicted and the actual MI classification accuracies, based on CSP-cv, CSP, CSSP, FBCSP, and BSSFO. The predicted performance had the highest *r*-squared with actual MI performance, based on CSP and FBCSP in Sessions 1 and 2, respectively (Session 1: *r*-squared = 0.31; Session 2: *r*-squared = 0.17). The lowest RMSE for actual MI performance was found under CSP-cv in both sessions (Session 1: RMSE = 13.79%; Session 2: RMSE = 14.55%).

**TABLE 9:** Relationship between actual MI performance and predicted MI performance using connectivity strength from SMA to right DLPFC.

**Classifier**	**Session 1**		**Session 2**	
	***r*-squared**	**RMSE (%)**	***r*-squared**	**RMSE (%)**
CSP_cv	0.28	13.79	0.11	14.55
CSP	0.31	15.78	0.10	16.73
CSSP	0.25	16.46	0.14	17.21
FBCSP	0.20	17.97	0.17	17.05
BSSFO	0.21	18.04	0.11	17.63

*CSP_cv, common spatial pattern with cross-validation; CSP, common spatial pattern; CSSP, common spatio-spectral pattern; FBCSP, filter bank common spatial pattern; BSSFO, Bayesian spatio-spectral filter optimization.*

## Discussion

In this study, we investigated coupling strength as a new correlate with MI-BCI performance, using the DCM of the resting-state EEG. The MI-BCI performance was predicted by measuring this coupling between brain regions. A connection from the SMA to right DLPFC in the high-MI group was observed to be significantly higher than in the low-MI group. Moreover, this connection showed a significantly positive correlation with MI performance in both sessions under five classifiers.

The MI involves a variety of brain regions and successfully performs the information exchange for the integration of relevant regions. Specifically, a resting-state network with an efficient exchange of information facilitates MI-BCI performance (Zhang et al., 2015b). Interestingly, our results show that the MI-BCI performance can be predicted using the coupling strength from the SMA to right DLPFC in resting-state EEG; during MI, the SMA and DLPFC exhibited observable activations (Mizuguchi et al., 2013). The SMA plays a central role in the preparation of behavior, and it acts as a high-level motor control prohibiting the execution of MI responses (Nachev et al., 2008; Kim et al., 2018). These findings have already been proved using the DCM (Kasess et al., 2008). The DLPFC has been reported to be involved in the early phases of motor training (Pascual-Leone et al., 1996). The frontal region is also affected by cognitive events, and it is responsible for motor planning and programming (Kim et al., 2018). In particular, the right DLPFC plays a crucial role in cognitive controls such as motor attention or inhibition (Mizuguchi et al., 2013). In fact, it is already reported that right DLPFC in the resting-state as a core region is correlated with MI-BCI performance (Zhang et al., 2016). During MI, coupling from SMA to DLPFC has been reported to play a critical role in the motor control needed to move a finger (Kim et al., 2018). As a result of fMRI, DLPFC was not connected to M1, but to SMA during MI (Mizuguchi et al., 2013). The SMA is causally connected into the DLPFC and this relates to higher-order cognitive motor processes such as motor control and preparation (O’Shea and Moran, 2017). Therefore, the coupling strength from the SMA to right DLPFC, which is already determined in the resting-state, affects brain activity during MI; it influences MI-BCI performance.

We observed changes in MI-BCI performance across different sessions with various classifiers. This was motivated by the variations observed in EEG (Lotte et al., 2007). We also confirmed that different coupling strengths correlated with MI-BCI performance (depending on the session or classifier), except for the connections from the SMA to right DLPFC. Although we did not observe a significant correlation with MI-BCI performance across both sessions, coupling strength from the left DLPFC to SMA, or from the left PMC to left DLPFC, may also be important. Because there is no actual movement taking place during MI, secondary motor areas such as the PMC and SMA are more relevant to MI than M1 (Park et al., 2015b). We found relations across several motor areas; however, none were observed for M1. Even in stroke patients, the activity of the SMA affects MI performance more than M1 (Park et al., 2015a).

The crucial relationship with MI-BCI performance was not found using band power and questionnaires. Some studies have reported that SMR such as mu rhythms in the resting-state is related to MI performance (Blankertz et al., 2010; Kwon et al., 2020). In fact, this argument seems plausible because alpha and beta power are decreased during the MI and used as typical features of the MI paradigm. However, other studies did not observe the significant correlation between spectral power and MI-BCI performance (Jeunet et al., 2015). Interestingly, even though it turns out to be an obvious consistent relationship between the MI-BCI performance and SMR, no significant correlation has been revealed. This is probably due to differences in experimental protocols. Previous studies presented SMR as a reliable predictor were instructed to imagine moving the hand itself. However, in other studies, the rotation of hand can be imagined. In other words, it is a different protocol. In our study, the subject imagines grasping their hands. Therefore, the resting-state SMR is clearly associated with brain changes during the MI, but their role as an MI-BCI predictor may vary depending on the experimental protocol. We also used the mu power based on a shorter baseline period, whereas the mu rhythm predictor in another study was computed as the maximum difference between the power spectral density and the estimated noise floor over the Laplace-filtered sensorimotor channels (Blankertz et al., 2010). In this regard, different ways of extracting mu rhythm are likely to have had these different observations. In addition, the brain activity—such as band power during resting-state—simply indicates the state of certain brain regions, but it cannot indicate the interregional relationships (Lee et al., 2017); these connections, in addition to the sensorimotor cortex associated with the motor network, seem significant (Sharma et al., 2006). In fact, resting-state connectivity is correlated with motor task performance (Gregory et al., 2014). Previous studies have investigated several networks enacting MI (Lorey et al., 2011). It appears that brain connectivity is more relevant to MI performance than the brain activity of certain regions. Furthermore, the questionnaires were too subjective because each subject had different criteria for predicting MI-BCI performance.

By applying our results to the BCI-inefficiency problem, it can be seen that a possible reason for BCI-inefficiency is that subjects have a less active motor network in the motor preparation regions related to cognitive processes in the resting-state (Ahn et al., 2013). Therefore, improvement of MI performance requires a new approach to activate the motor network. Performing upper extremity exercises is a good way to activate a motor network during the resting-state (Ma et al., 2011). In stroke patients, upper extremity rehabilitation has been shown to activate the resting-state effective connectivity of the motor network (Andrew James et al., 2009). This implies that MI-BCI performance can be improved by enhancing the connectivity strengths associated with motor planning, such as the coupling strength from the SMA to right DLPFC. Therefore, if subjects had been asked about their exercise habits in the pre-experimental questionnaire, the responses may have shown some correlation with MI performance. As another approach, the resting-state motor network can be improved by the direct stimulation of the brain, through transcranial direct current stimulation or transcranial magnetic stimulation (Fischer et al., 2017).

This study had a few limitations. First, we did not check whether ME performance was predictable using our proposed coupling strength. ME and MI share a common mechanism and motor circuit-related motor network (Lee et al., 2016; Daeglau et al., 2020). In this regard, it would have been more effective to examine the relationship with ME performance, to enable the wider use of the predictor in the future. Second, we used all brain regions when finding predictors through the DCM. However, measuring the entire brain is impractical. Therefore, based on our results, we need to use only a small number of EEG channels to predict MI-BCI performance in the future. Third, we used only grasping imagery, and brain activity is known to vary depending on the type of action. For example, the SMA activity depends on whether it executes a large movement (e.g., wrist movements of hand rotation) or a small movement (e.g., finger movements in hand grasping) (Park et al., 2015b). Therefore, it is necessary to apply them accordingly to different actions. Last, computation time is very important in real applications. The model and specific ROIs are already selected, so we can measure the proposed strength in sec. Nevertheless, we did not directly compare the existing paper with the computation time. Therefore, in the future, it is necessary to compare the computing time for this practicality.

## Conclusion

We proposed an MI-BCI predictor from the resting-state EEG using DCM. Our study is valuable in two ways. The first is its investigation of the effective connectivity (with directionality) related to MI performance; it facilitates a more analytical understanding of why the performance is lower in low-MI groups. Our results suggest that for subjects with “BCI-inefficiency,” appropriate alternatives can be implemented to improve MI-BCI performance. Second, we show the possibility of predicting MI performance using predictors measured before the time-consuming MI-BCI experiment takes place. Therefore, our predictor can be used to sort out BCI-inefficiency before subjects perform a task in the real application. This can help prevent the unnecessary waste of time and resources when implementing MI-BCI in practice.

## Data Availability Statement

The datasets generated for this study are available on request to the corresponding author.

## Ethics Statement

The studies involving human participants were reviewed and approved by Korea University Institutional Review Board (1040548-KUIRB-16-159-A-2). The patients/participants provided their written informed consent to participate in this study.

## Author Contributions

ML, J-GY, and S-WL designed the experiments. ML and J-GY analyzed the data and drafted the manuscript. ML and S-WL critically revised the manuscript and contributed important intellectual comments. All authors contributed to the article and approved the submitted version.

## Conflict of Interest

The authors declare that the research was conducted in the absence of any commercial or financial relationships that could be construed as a potential conflict of interest.
