# Supplementary material for: Predicting Motor Imagery Performance From Resting-State EEG Using Dynamic Causal Modeling
Source: Front Hum Neurosci. 2020 Aug 6;14:321. doi: 10.3389/fnhum.2020.00321 (PMC7438792; doi:10.3389/fnhum.2020.00321)
Supplement: Supplementary file 1 [file Data_Sheet_1.docx]

Supplementary Material

Predicting Motor Imagery Performance From Resting-State EEG Using Dynamic Causal Modeling

Minji Lee^1¶^, Jae-Geun Yoon^1¶^, and Seong-Whan Lee^2*^

^1^Department of Brain and Cognitive Engineering, Korea University, Seoul, Republic of Korea
^2^Department of Artificial Intelligence, Korea University, Seoul, Republic of Korea

^¶^These authors contributed equally to this work.

*** Correspondence:**Seong-Whan Lee
sw.lee@korea.ac.kr

# Supplementary Figures and Tables

## Supplementary Figures


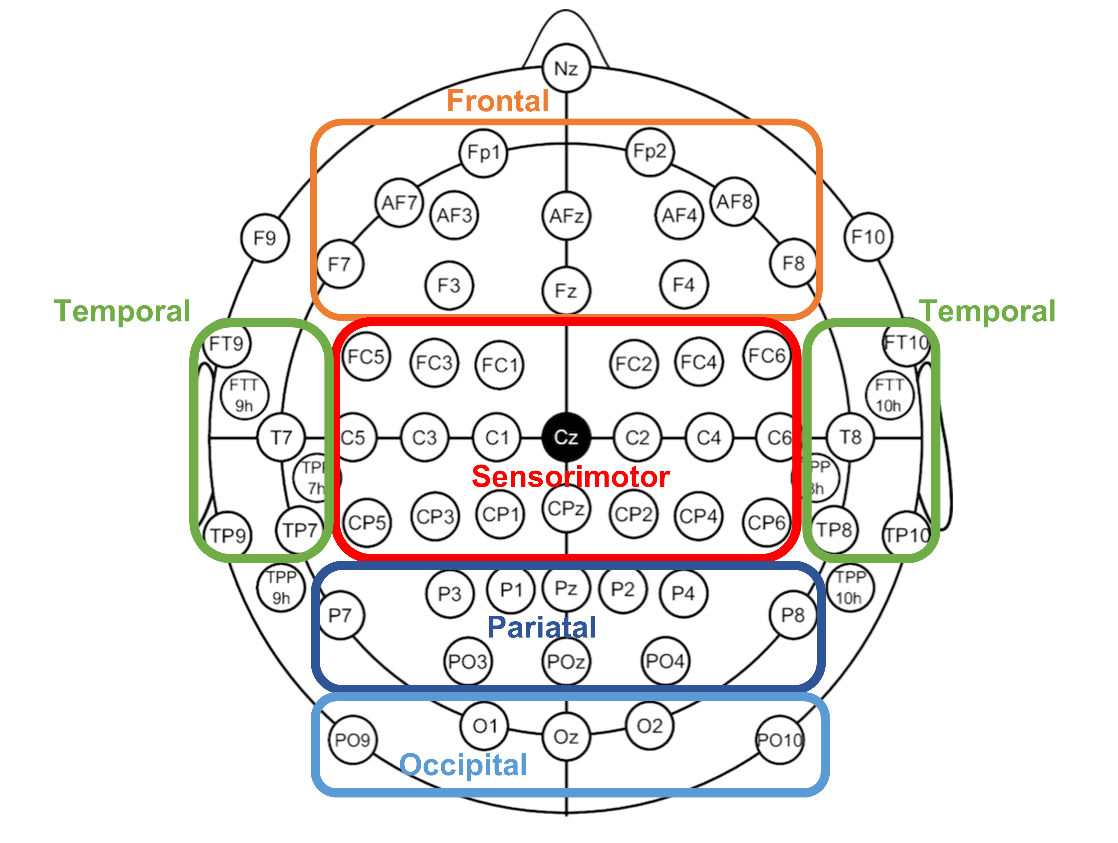


**Supplementary Figure S1**. **The channel information.** We divided five brain regions into frontal, sensorimotor, temporal, parietal, and occipital regions.


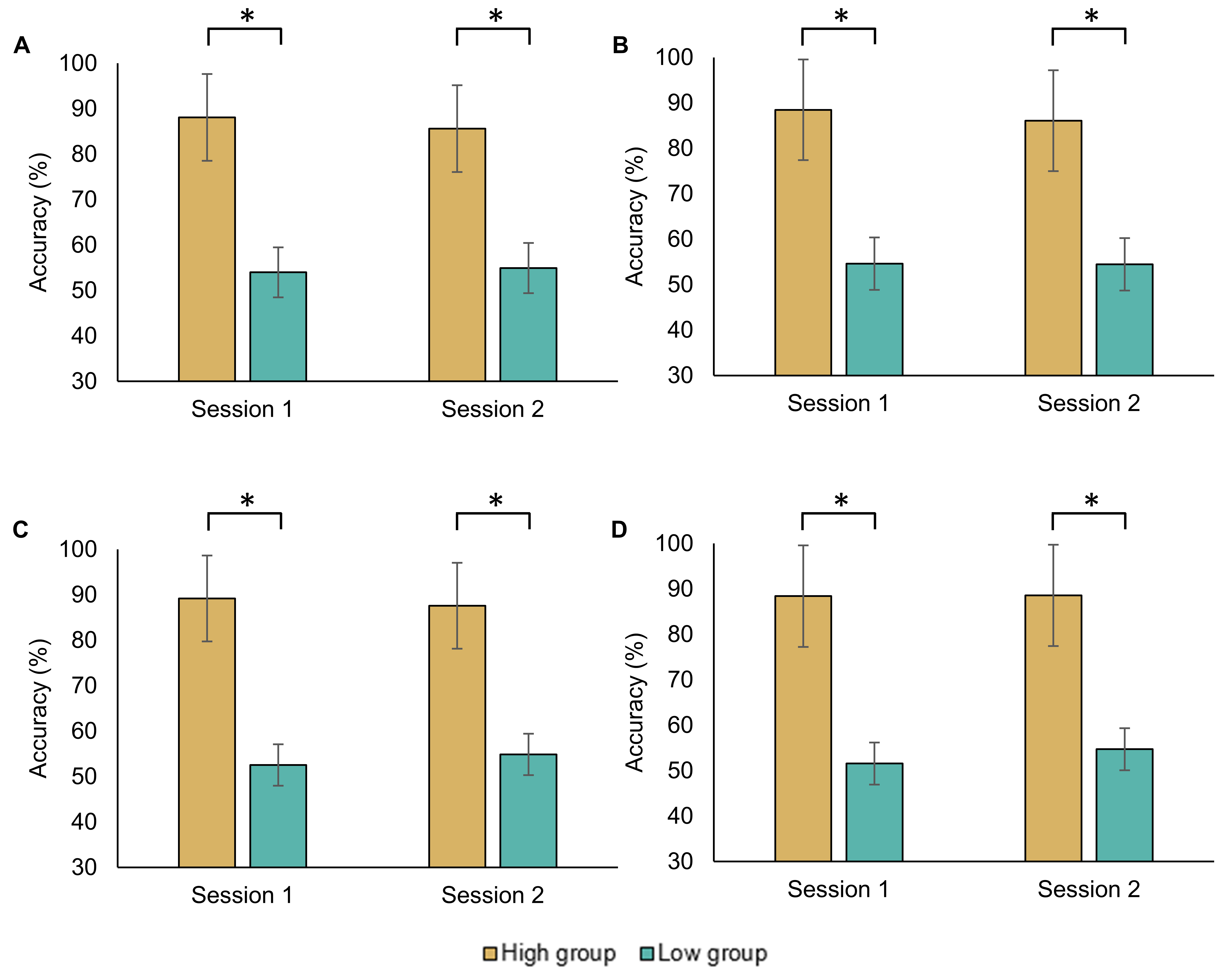


**Supplementary Figure S2. Comparison of averaged MI classification accuracy between high- and low-MI performance groups based on (A) CSP, (B) CSSP, (C) FBCSP, and (D) BSSFO.** The group was divided based on the median of MI classification accuracy across all subjects. The p-values below 0.05 are highlighted by an asterisk. CSP = common spatial pattern, CSSP = common spatio-spectral pattern, FBCSP = filter bank common spatial pattern, BSSFO = Bayesian spatio-spectral filter optimization.


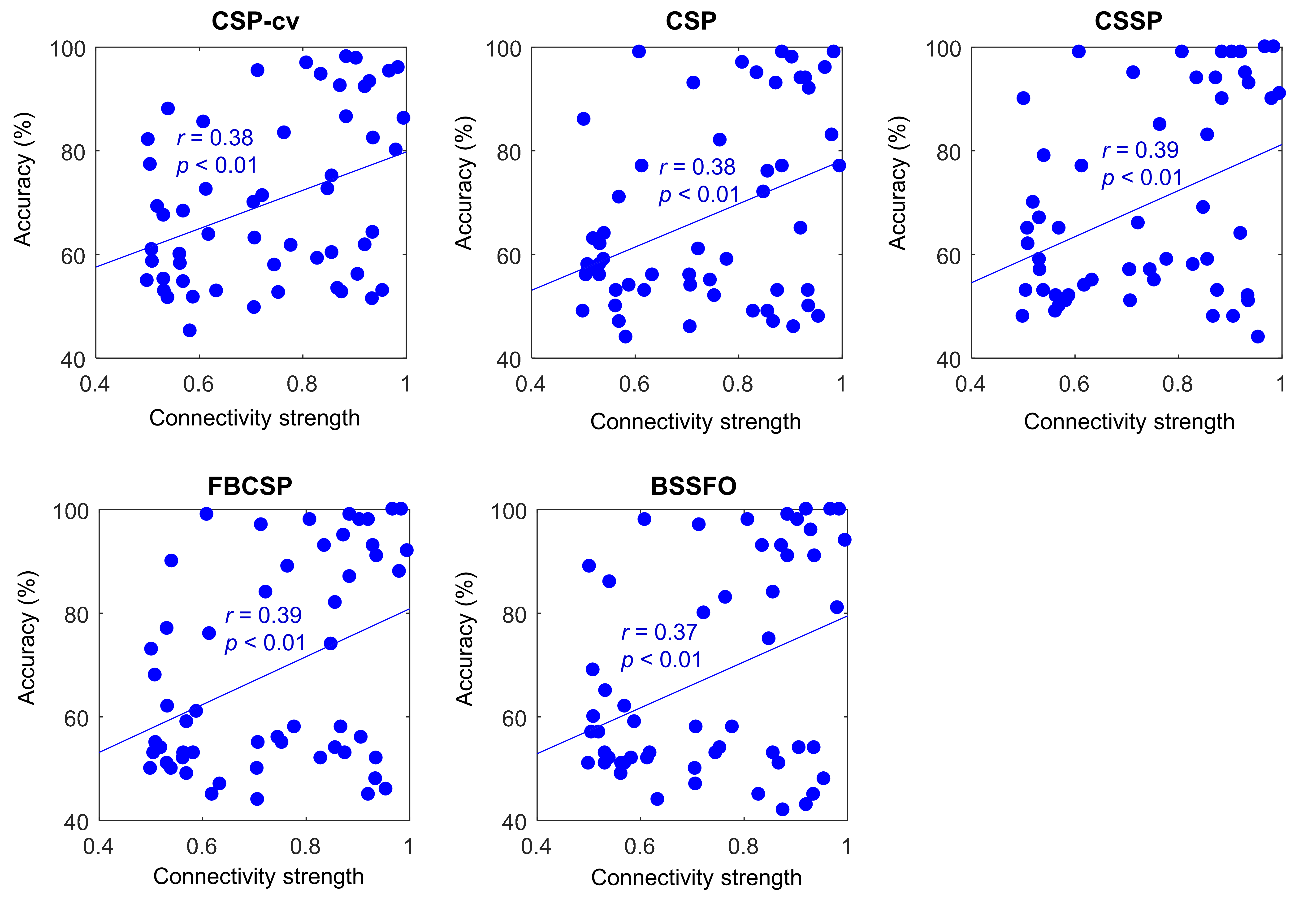


**Supplementary Figure S3. Correlation between connectivity strength from the left DLPFC to SMA and MI-BCI performance.** Each colored dot represents an individual connectivity strength from the left DLPFC to SMA and MI performance in Session 1. SMA = supplementary motor area, DLPFC = dorsolateral prefrontal cortex, CSP-cv = common spatial pattern with cross-validation, CSP = common spatial pattern, CSSP = common spatio-spectral pattern, FBCSP = filter bank common spatial pattern, BSSFO = Bayesian spatio-spectral filter optimization.

## Supplementary Tables

**Supplementary Table S1.** **The statistical differences in effective connectivity between high- and low-MI performance groups based on CSP.** In Session 1, Model 4 is selected and there are 20 connections. In Session 2, Model 2 is selected and there are 16 connections. Therefore, four connections in Session 2 are excluded (‘NaN’). The session factor indicates Session 1 and Session 2, whereas the group factor indicates high-MI performance group and low-MI performance group. The session × group represents the interaction between session and group factors. The p-values below 0.05 are highlighted in bold. ROI = region of interest, dof = degree of freedom, l/rM1 = left/right primary motor cortex, l/rPMC = left/right pre-motor cortex, l/rDLPFC = left/right dorsolateral prefrontal cortex, SMA = supplementary motor area.

| ROI | | Session | | | Group | | | Session × Group | | |
| --- | --- | --- | --- | --- | --- | --- | --- | --- | --- | --- |
| From | To | *dof* | *F* | *p-value* | *dof* | *F* | *p-value* | *dof* | *F* | *p-value* |
| lM1 | SMA | 1 | 1.28 | 0.260 | 1 | 0.39 | 0.535 | 1 | 0.41 | 0.521 |
| rM1 | SMA | 1 | 0.57 | 0.453 | 1 | 0.10 | 0.751 | 1 | 0.49 | 0.484 |
| lPMC | SMA | 1 | 1.80 | 0.182 | 1 | 1.74 | 0.189 | 1 | 0.08 | 0.779 |
| rPMC | SMA | 1 | 1.37 | 0.244 | 1 | 0.16 | 0.692 | 1 | 0.60 | 0.440 |
| lDLPFC | SMA | 0 | 0 | NaN | 1 | 11.58 | **0.001** | 0 | 0 | NaN |
| rDLPFC | SMA | 0 | 0 | NaN | 1 | 0.19 | 0.662 | 0 | 0 | NaN |
| SMA | lM1 | 0 | 0 | NaN | 1 | 2.75 | 0.103 | 0 | 0 | NaN |
| rM1 | lM1 | 1 | 3.27 | 0.073 | 1 | 1.84 | 0.177 | 1 | 0.72 | 0.398 |
| lPMC | lM1 | 1 | 1.38 | 0.243 | 1 | 0.51 | 0.474 | 1 | 0.98 | 0.324 |
| SMA | rM1 | 0 | 0 | NaN | 1 | 0.01 | 0.940 | 0 | 0 | NaN |
| lM1 | rM1 | 1 | 0.92 | 0.338 | 1 | 0.11 | 0.740 | 1 | 0.01 | 0.932 |
| rPMC | rM1 | 1 | 0.88 | 0.350 | 1 | 0.86 | 0.355 | 1 | 0.04 | 0.847 |
| rPMC | lPMC | 1 | 2.98 | 0.087 | 1 | 0.85 | 0.360 | 1 | 1.03 | 0.312 |
| lPMC | rPMC | 1 | 0.39 | 0.531 | 1 | 0.48 | 0.490 | 1 | 0.31 | 0.576 |
| SMA | lDLPFC | 1 | 0.42 | 0.517 | 1 | 0.13 | 0.719 | 1 | 0.40 | 0.527 |
| lPMC | lDLPFC | 1 | 0.16 | 0.690 | 1 | 4.12 | **0.045** | 1 | 2.42 | 0.122 |
| rDLPFC | lDLPFC | 1 | 0.33 | 0.567 | 1 | 0.87 | 0.353 | 1 | 0.42 | 0.517 |
| SMA | rDLPFC | 1 | 0.45 | 0.502 | 1 | 18.71 | **<0.001** | 1 | 0.01 | 0.928 |
| rPMC | rDLPFC | 1 | 0.24 | 0.621 | 1 | 0.05 | 0.819 | 1 | 1.97 | 0.162 |
| lDLPFC | rDLPFC | 1 | 1.42 | 0.236 | 1 | 0.10 | 0.7555 | 1 | 0.13 | 0.715 |

**Supplementary Table S2.** **The statistical differences in effective connectivity between high- and low-MI performance groups based on CSSP.** In Session 1, Model 4 is selected and there are 20 connections. In Session 2, Model 2 is selected and there are 16 connections. Therefore, four connections in Session 2 are excluded (‘NaN’). The session factor indicates Session 1 and Session 2, whereas the group factor indicates high-MI performance group and low-MI performance group. The session × group represents the interaction between session and group factors. The p-values below 0.05 are highlighted in bold. ROI = region of interest, dof = degree of freedom, l/rM1 = left/right primary motor cortex, l/rPMC = left/right pre-motor cortex, l/rDLPFC = left/right dorsolateral prefrontal cortex, SMA = supplementary motor area.

| ROI | | Session | | | Group | | | Session × Group | | |
| --- | --- | --- | --- | --- | --- | --- | --- | --- | --- | --- |
| From | To | *dof* | *F* | *p-value* | *dof* | *F* | *p-value* | *dof* | *F* | *p-value* |
| lM1 | SMA | 1 | 2.00 | 0.160 | 1 | 0.65 | 0.423 | 1 | 0.63 | 0.428 |
| rM1 | SMA | 1 | 0.42 | 0.520 | 1 | 0.02 | 0.892 | 1 | 0.78 | 0.378 |
| lPMC | SMA | 1 | 2.04 | 0.156 | 1 | 1.37 | 0.243 | 1 | 0.10 | 0.750 |
| rPMC | SMA | 1 | 1.14 | 0.287 | 1 | 0.32 | 0.573 | 1 | 0.09 | 0.762 |
| lDLPFC | SMA | 0 | 0 | NaN | 1 | 7.00 | **0.010** | 0 | 0 | NaN |
| rDLPFC | SMA | 0 | 0 | NaN | 1 | 0.07 | 0.797 | 0 | 0 | NaN |
| SMA | lM1 | 0 | 0 | NaN | 1 | 0.20 | 0.652 | 0 | 0 | NaN |
| rM1 | lM1 | 1 | 3.01 | 0.085 | 1 | 2.58 | 0.111 | 1 | 0.41 | 0.521 |
| lPMC | lM1 | 1 | 1.54 | 0.217 | 1 | 0.89 | 0.347 | 1 | 1.53 | 0.218 |
| SMA | rM1 | 0 | 0 | NaN | 1 | 0.01 | 0.912 | 0 | 0 | NaN |
| lM1 | rM1 | 1 | 1.03 | 0.311 | 1 | 0.26 | 0.611 | 1 | 0.18 | 0.668 |
| rPMC | rM1 | 1 | 0.78 | 0.379 | 1 | 1.59 | 0.209 | 1 | 0.40 | 0.529 |
| rPMC | lPMC | 1 | 2.60 | 0.109 | 1 | 0.46 | 0.498 | 1 | 2.38 | 0.125 |
| lPMC | rPMC | 1 | 0.47 | 0.495 | 1 | 0.46 | 0.500 | 1 | 0.26 | 0.609 |
| SMA | lDLPFC | 1 | 0.43 | 0.511 | 1 | 0.35 | 0.555 | 1 | 0.33 | 0.565 |
| lPMC | lDLPFC | 1 | 0.52 | 0.472 | 1 | 3.91 | 0.050 | 1 | 4.63 | **0.033** |
| rDLPFC | lDLPFC | 1 | 0.06 | 0.807 | 1 | 0.11 | 0.744 | 1 | 3.17 | 0.077 |
| SMA | rDLPFC | 1 | 0.73 | 0.395 | 1 | 22.65 | **<0.001** | 1 | 0.05 | 0.816 |
| rPMC | rDLPFC | 1 | 0.10 | 0.745 | 1 | 0.01 | 0.935 | 1 | 0.02 | 0.899 |
| lDLPFC | rDLPFC | 1 | 1.40 | 0.239 | 1 | 0.01 | 0.979 | 1 | 0.02 | 0.895 |

**Supplementary Table S3.** **The statistical differences in effective connectivity between high- and low-MI performance groups based on FBCSP.** In Session 1, Model 4 is selected and there are 20 connections. In Session 2, Model 2 is selected and there are 16 connections. Therefore, four connections in Session 2 are excluded (‘NaN’). The session factor indicates Session 1 and Session 2, whereas the group factor indicates high-MI performance group and low-MI performance group. The session × group represents the interaction between session and group factors. The p-values below 0.05 are highlighted in bold. ROI = region of interest, dof = degree of freedom, l/rM1 = left/right primary motor cortex, l/rPMC = left/right pre-motor cortex, l/rDLPFC = left/right dorsolateral prefrontal cortex, SMA = supplementary motor area.

| ROI | | Session | | | Group | | | Session × Group | | |
| --- | --- | --- | --- | --- | --- | --- | --- | --- | --- | --- |
| From | To | *dof* | *F* | *p-value* | *dof* | *F* | *p-value* | *dof* | *F* | *p-value* |
| lM1 | SMA | 1 | 1.49 | 0.225 | 1 | 0.01 | 0.931 | 1 | 0.13 | 0.721 |
| rM1 | SMA | 1 | 0.75 | 0.388 | 1 | 0.01 | 0.998 | 1 | 0.05 | 0.823 |
| lPMC | SMA | 1 | 1.71 | 0.193 | 1 | 0.65 | 0.420 | 1 | 0.35 | 0.554 |
| rPMC | SMA | 1 | 1.09 | 0.298 | 1 | 0.24 | 0.623 | 1 | 0.01 | 0.904 |
| lDLPFC | SMA | 0 | 0 | NaN | 1 | 7.97 | **0.006** | 0 | 0 | NaN |
| rDLPFC | SMA | 0 | 0 | NaN | 1 | 0.14 | 0.714 | 0 | 0 | NaN |
| SMA | lM1 | 0 | 0 | NaN | 1 | 0.46 | 0.498 | 0 | 0 | NaN |
| rM1 | lM1 | 1 | 2.70 | 0.103 | 1 | 6.05 | **0.015** | 1 | 0.05 | 0.828 |
| lPMC | lM1 | 1 | 1.37 | 0.244 | 1 | 1.16 | 0.283 | 1 | 1.61 | 0.207 |
| SMA | rM1 | 0 | 0 | NaN | 1 | 0.01 | 0.940 | 0 | 0 | NaN |
| lM1 | rM1 | 1 | 0.76 | 0.384 | 1 | 0.32 | 0.570 | 1 | 0.14 | 0.705 |
| rPMC | rM1 | 1 | 0.87 | 0.352 | 1 | 0.89 | 0.348 | 1 | 0.52 | 0.470 |
| rPMC | lPMC | 1 | 3.10 | 0.081 | 1 | 0.08 | 0.776 | 1 | 1.59 | 0.209 |
| lPMC | rPMC | 1 | 0.41 | 0.522 | 1 | 0.03 | 0.854 | 1 | 0.09 | 0.758 |
| SMA | lDLPFC | 1 | 0.44 | 0.510 | 1 | 0.01 | 0.966 | 1 | 0.58 | 0.446 |
| lPMC | lDLPFC | 1 | 0.24 | 0.626 | 1 | 1.41 | 0.237 | 1 | 1.38 | 0.243 |
| rDLPFC | lDLPFC | 1 | 0.22 | 0.641 | 1 | 0.81 | 0.371 | 1 | 0.84 | 0.361 |
| SMA | rDLPFC | 1 | 1.10 | 0.296 | 1 | 18.33 | **<0.001** | 1 | 0.21 | 0.646 |
| rPMC | rDLPFC | 1 | 0.09 | 0.768 | 1 | 0.09 | 0.761 | 1 | 0.01 | 0.967 |
| lDLPFC | rDLPFC | 1 | 1.50 | 0.223 | 1 | 0.18 | 0.674 | 1 | 0.01 | 0.991 |

**Supplementary Table S4.** **The statistical differences in effective connectivity between high- and low-MI performance groups based on BSSFO.** In Session 1, Model 4 is selected and there are 20 connections. In Session 2, Model 2 is selected and there are 16 connections. Therefore, four connections in Session 2 are excluded (‘NaN’). The session factor indicates Session 1 and Session 2, whereas the group factor indicates high-MI performance group and low-MI performance group. The session × group represents the interaction between session and group factors. The p-values below 0.05 are highlighted in bold. ROI = region of interest, dof = degree of freedom, l/rM1 = left/right primary motor cortex, l/rPMC = left/right pre-motor cortex, l/rDLPFC = left/right dorsolateral prefrontal cortex, SMA = supplementary motor area.

| ROI | | Session | | | Group | | | Session × Group | | |
| --- | --- | --- | --- | --- | --- | --- | --- | --- | --- | --- |
| From | To | *dof* | *F* | *p-value* | *dof* | *F* | *p-value* | *dof* | *F* | *p-value* |
| lM1 | SMA | 1 | 2.01 | 0.159 | 1 | 1.68 | 0.198 | 1 | 0.91 | 0.343 |
| rM1 | SMA | 1 | 1.06 | 0.306 | 1 | 0.05 | 0.817 | 1 | 1.39 | 0.241 |
| lPMC | SMA | 1 | 1.55 | 0.216 | 1 | 0.54 | 0.466 | 1 | 0.65 | 0.420 |
| rPMC | SMA | 1 | 1.10 | 0.297 | 1 | 0.10 | 0.752 | 1 | 0.01 | 0.912 |
| lDLPFC | SMA | 0 | 0 | NaN | 1 | 4.77 | **0.033** | 0 | 0 | NaN |
| rDLPFC | SMA | 0 | 0 | NaN | 1 | 0.03 | 0.861 | 0 | 0 | NaN |
| SMA | lM1 | 0 | 0 | NaN | 1 | 0.56 | 0.456 | 0 | 0 | NaN |
| rM1 | lM1 | 1 | 2.96 | 0.088 | 1 | 3.32 | 0.071 | 1 | 0.03 | 0.560 |
| lPMC | lM1 | 1 | 1.10 | 0.297 | 1 | 0.65 | 0.420 | 1 | 0.14 | 0.707 |
| SMA | rM1 | 0 | 0 | NaN | 1 | 0.24 | 0.624 | 0 | 0 | NaN |
| lM1 | rM1 | 1 | 0.61 | 0.437 | 1 | 0.01 | 0.841 | 1 | 0.77 | 0.381 |
| rPMC | rM1 | 1 | 0.96 | 0.328 | 1 | 2.45 | 0.120 | 1 | 0.04 | 0.842 |
| rPMC | lPMC | 1 | 3.89 | 0.051 | 1 | 0.36 | 0.549 | 1 | 0.10 | 0.753 |
| lPMC | rPMC | 1 | 0.39 | 0.534 | 1 | 0.05 | 0.822 | 1 | 0.04 | 0.845 |
| SMA | lDLPFC | 1 | 0.33 | 0.564 | 1 | 0.22 | 0.639 | 1 | 1.26 | 0.264 |
| lPMC | lDLPFC | 1 | 0.16 | 0.691 | 1 | 0.02 | 0.886 | 1 | 0.30 | 0.586 |
| rDLPFC | lDLPFC | 1 | 0.21 | 0.651 | 1 | 0.01 | 0.997 | 1 | 0.76 | 0.385 |
| SMA | rDLPFC | 1 | 1.02 | 0.315 | 1 | 15.47 | **<0.001** | 1 | 0.46 | 0.499 |
| rPMC | rDLPFC | 1 | 0.07 | 0.789 | 1 | 0.55 | 0.458 | 1 | 0.01 | 0.907 |
| lDLPFC | rDLPFC | 1 | 1.08 | 0.300 | 1 | 0.010 | 0.755 | 1 | 1.18 | 0.279 |

**Supplementary Table S5. Correlation between connectivity strength and MI classification accuracy.** In Session 1, Model 4 is selected and there are 20 connections. In Session 2, Model 2 is selected and there are 16 connections. Therefore, four connections in Session 2 are excluded (‘NaN’). The *p*-values below 0.05 are highlighted in bold. ROI = region of interest, CSP = common spatial pattern, CSSP = common spatio-spectral pattern, FBCSP = filter bank common spatial pattern, BSSFO = Bayesian spatio-spectral filter optimization, l/rM1 = left/right primary motor cortex, l/rPMC = left/right pre-motor cortex, l/rDLPFC = left/right dorsolateral prefrontal cortex, SMA = supplementary motor area.

| **ROI** | | **CSP** | | | | **CSSP** | | | | **FBCSP** | | | | **BSSFO** | | | |
| --- | --- | --- | --- | --- | --- | --- | --- | --- | --- | --- | --- | --- | --- | --- | --- | --- | --- |
|  |  | **Session 1** | | **Session 2** | | **Session1** | | **Session 2** | | **Session 1** | | **Session 2** | | **Session 1** | | **Session 2** | |
| *From* | *To* | *r-value* | *p-value* | *r-value* | *p-value* | *r-value* | *p-value* | *r-value* | *p-value* | *r-value* | *p-value* | *r-value* | *p-value* | *r-value* | *p-value* | *r-value* | *p-value* |
| lM1 | SMA | 0.096 | 0.488 | 0.066 | 0.638 | 0.045 | 0.749 | 0.090 | 0.519 | 0.105 | 0.449 | 0.002 | 0.990 | 0.093 | 0.505 | 0.151 | 0.277 |
| rM1 | SMA | 0.031 | 0.826 | 0.018 | 0.898 | 0.002 | 0.986 | 0.0430 | 0.759 | -0.017 | 0.901 | 0.084 | 0.545 | -0.097 | 0.484 | 0.103 | 0.458 |
| lPMC | SMA | -0.109 | 0.433 | -0.129 | 0.353 | -0.150 | 0.278 | -0.130 | 0.348 | -0.117 | 0.399 | -0.110 | 0.430 | -0.168 | 0.226 | -0.121 | 0.385 |
| rPMC | SMA | -0.036 | 0.795 | -0.016 | 0.909 | -0.068 | 0.624 | -0.079 | 0.571 | -0.086 | 0.536 | -0.031 | 0.822 | -0.052 | 0.710 | -0.010 | 0.942 |
| lDLPFC | SMA | 0.379 | **0.005** | NaN | NaN | 0.391 | **0.003** | NaN | NaN | 0.390 | **0.004** | NaN | NaN | 0.365 | **0.007** | NaN | NaN |
| rDLPFC | SMA | -0.105 | 0.450 | NaN | NaN | -0.054 | 0.700 | NaN | NaN | -0.021 | 0.880 | NaN | NaN | -0.047 | 0.734 | NaN | NaN |
| SMA | lM1 | -0.166 | 0.230 | NaN | NaN | -0.104 | 0.453 | NaN | NaN | -0.114 | 0.411 | NaN | NaN | -0.156 | 0.260 | NaN | NaN |
| rM1 | lM1 | -0.214 | 0.121 | -0.075 | 0.591 | -0.230 | 0.094 | -0.107 | 0.439 | -0.296 | **0.030** | -0.134 | 0.333 | -0.282 | **0.039** | -0.076 | 0.584 |
| lPMC | lM1 | -0.131 | 0.344 | -0.029 | 0.834 | -0.155 | 0.263 | -0.046 | 0.742 | -0.148 | 0.286 | -0.057 | 0.683 | -0.141 | 0.309 | -0.079 | 0.572 |
| SMA | rM1 | 0.061 | 0.659 | NaN | NaN | 0.026 | 0.854 | NaN | NaN | -0.035 | 0.801 | NaN | NaN | -0.002 | 0.988 | NaN | NaN |
| lM1 | rM1 | -0.057 | 0.680 | -0.043 | 0.760 | -0.048 | 0.733 | -0.090 | 0.517 | -0.015 | 0.913 | -0.040 | 0.774 | -0.024 | 0.864 | -0.038 | 0.783 |
| rPMC | rM1 | 0.140 | 0.313 | 0.066 | 0.637 | 0.150 | 0.280 | -0.011 | 0.938 | 0.118 | 0.395 | 0.068 | 0.623 | 0.094 | 0.499 | 0.074 | 0.594 |
| rPMC | lPMC | 0.213 | 0.122 | -0.023 | 0.867 | 0.202 | 0.142 | -0.098 | 0.482 | 0.137 | 0.322 | -0.061 | 0.660 | 0.171 | 0.215 | 0.078 | 0.577 |
| lPMC | rPMC | 0.085 | 0.540 | 0.019 | 0.894 | 0.032 | 0.818 | -0.006 | 0.966 | -0.035 | 0.803 | 0.031 | 0.824 | 0.026 | 0.854 | 0.010 | 0.942 |
| SMA | lDLPFC | 0.120 | 0.388 | -0.021 | 0.881 | 0.057 | 0.685 | -0.074 | 0.593 | 0.072 | 0.607 | -0.100 | 0.471 | 0.102 | 0.463 | -0.111 | 0.423 |
| lPMC | lDLPFC | -0.079 | 0.569 | -0.320 | **0.018** | -0.082 | 0.556 | -0.306 | **0.024** | -0.019 | 0.894 | -0.287 | **0.035** | 0.024 | 0.864 | -0.212 | 0.124 |
| rDLPFC | lDLPFC | 0.093 | 0.504 | -0.274 | **0.045** | 0.078 | 0.574 | -0.241 | 0.079 | 0.041 | 0.769 | -0.197 | 0.153 | 0.108 | 0.437 | -0.156 | 0.260 |
| SMA | rDLPFC | 0.540 | **<0.001** | 0.374 | **0.005** | 0.514 | **<0.001** | 0.372 | **0.006** | 0.484 | **<0.001** | 0.407 | **0.002** | 0.490 | **<0.001** | 0.400 | **0.003** |
| rPMC | rDLPFC | -0.053 | 0.702 | 0.137 | 0.324 | -0.040 | 0.776 | 0.090 | 0.518 | -0.001 | 0.992 | 0.143 | 0.302 | -0.032 | 0.820 | 0.144 | 0.297 |
| lDLPFC | rDLPFC | 0.056 | 0.688 | -0.030 | 0.827 | 0.011 | 0.963 | -0.016 | 0.909 | 0.001 | 0.994 | -0.042 | 0.762 | 0.049 | 0.722 | -0.064 | 0.644 |
